# Circadian Dysrhythmias, Physiological Aberrations, and the Link to Skin Cancer

**DOI:** 10.3390/ijms17050621

**Published:** 2016-04-26

**Authors:** Daniel Gutierrez, Joshua Arbesman

**Affiliations:** 1 School of Medicine, Case Western Reserve University, Cleveland, OH 44106, USA; dxg290@case.edu; 2 Department of Dermatology, University Hospitals Case Medical Center, Cleveland, OH 44106, USA

**Keywords:** circadian rhythms, skin cancer, melatonin, UVR, epidemiology

## Abstract

Circadian rhythms are core regulators of a variety of mammalian physiologic processes and oscillate in a 24-h pattern. Many peripheral organs possess endogenous rhythmicity that is then modulated by a master clock; the skin is one of these peripheral organs. The dysregulation of rhythms is associated with decreased ability to ameliorate cellular stressors at a local and global level, which then increases the propensity for the development of neoplastic growths. In this article, we review the implications of altered circadian rhythms on DNA repair as well as modified gene expression of core clock proteins with particular focus on skin models. These findings are then correlated with epidemiologic data regarding skin cancer to showcase the effects of circadian disruption on this phenomenon.

## 1. Introduction to Circadian Rhythms

Circadian rhythms are self-sustaining, endogenous processes that display oscillatory patterns of roughly 24 h that include the sleep-wake cycle as well as fluctuations in hormone production [1]. These rhythms are generated, regulated, and maintained by the circadian time-keeping system, or circadian clock. This consists of the following components: the central oscillator that intrinsically keeps the rhythm, input pathways that receive information from environmental stimuli, and output pathways to exert neurohormonal influence on a diverse array of biological and behavioral events [1,2]. The suprachiasmatic nucleus (SCN) of the anterior hypothalamus performs the primary oversight of mammalian circadian rhythm and influences a variety of tissues in the human body [3,4]. While peripheral rhythmicity is present in most tissues [5], they remain under the strong influence of the SCN [4,6]. The skin is one such organ which has an endogenous circadian clock heavily modulated by the SCN. In this article, we will discuss the basic regulatory mechanisms of core circadian loop systems, clinical phenotypes resulting from genetic studies involving the clock machinery, and the implications of shift work, and consequently circadian rhythm dysrhythmia, on carcinogenicity as showcased through epidemiological studies. In addition, the role of circadian-influenced melatonin synthesis and DNA repair mechanisms on tumorigenesis will be explored. Finally, we will examine the expression of core clock proteins in tumor biopsies.

## 2. The Molecular Circadian Clock Machinery

**Figure 1:**
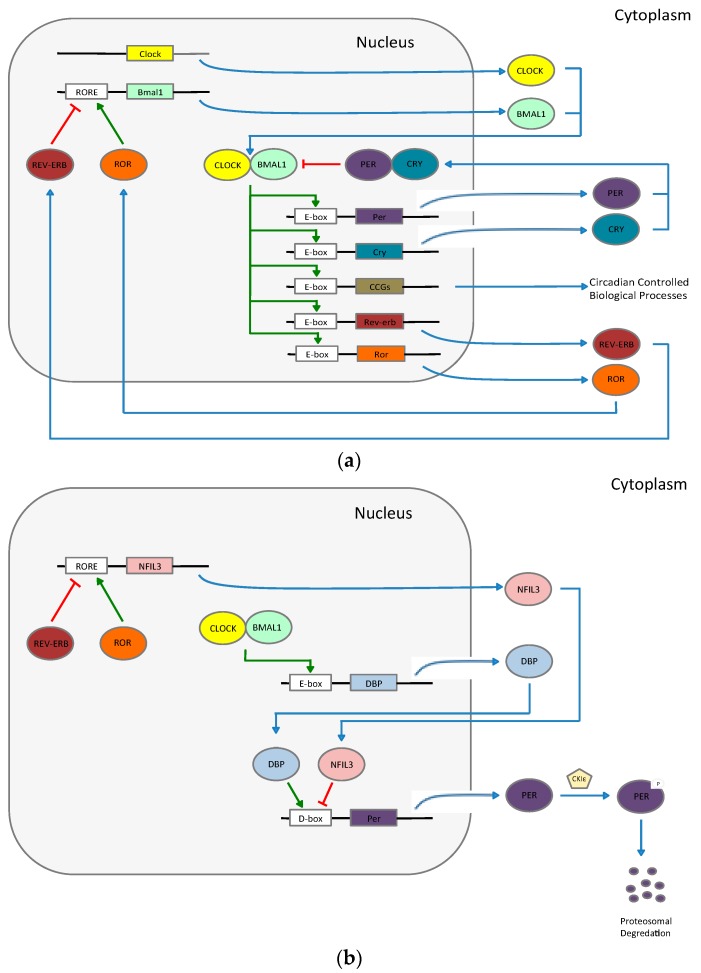
(**a**) A schematic of mammalian molecular clock demonstrating transcriptional-translational feedback loops. Brain and muscle ARNT-like protein 1 (BMAL1)-Circadian Locomotor Output Cycles Kaput (CLOCK) heterodimers positively regulate downstream products while Period (PER)-Cryptochrome (CRY) heterodimers negatively regulate downstream products. REV-ERB proteins enhance BMAL1 synthesis while Retinoic acid receptor-related orphan receptors (ROR) proteins repress BMAL1 synthesis. Blue arrows represent protein movements. Green arrows represent activation. Red arrows represent repression; (**b**) A schematic of mammalian molecular clock demonstrating transcriptional-translational feedback loops. D-box controlled protein production is repressed by nuclear factor interleukin-3 (NFIL3) and enhanced by albumin D-box binding protein (DBP). Posttranslational modification of core clock proteins helps to signal their degradation by proteasome complexes to control circadian rhythm. Blue arrows represent protein movements. Green arrows represent activation. Red arrows represent repression.

The molecular components of circadian rhythms are complex and comprised of both positive and negative transcriptional and translational feedback loops. In brief, we will summarize the protein pathways involved in the maintenance of a stable circadian rhythm. As explained by Curtis *et al.* [7] and Markova-Car [8], there are at least three distinct feedback loops that exist within the SCN (Figure 1a,b). The central loop starts with production of two critical proteins: brain and muscle ARNT-like protein 1 (BMAL1) and Circadian Locomotor Output Cycles Kaput (CLOCK). These two proteins heterodimerize and bind to the E-box elements on *Period* (*Per*) 1–3 and *Cryptochrome* (*Cry*) 1–2 genes to induce production of their protein products. The PER and CRY proteins themselves heterodimerize, return to the nucleus, and bind the BMAL1-CLOCK heterodimer to repress production of new PER and CRY proteins.

Another loop consists of the retinoic acid receptor-related orphan receptors (ROR) α, β, and γ and the REV-ERB α and β proteins; these proteins mediate *Bmal1* transcription. The BMAL1-CLOCK complex induces production of the ROR and REV-ERB proteins by binding to the E-box element in their promoter regions. Subsequently, these proteins (ROR and REV-ERB) competitively bind to the ROR response elements (RORE) in the *Bmal1* promoter region. Again, positive and negative feedback loops are established as ROR proteins upregulate transcription while REV-ERB proteins downregulate transcription of *Bmal1*.

The third feedback loop involves two factors that control production of D-box proteins and provide an additional component of regulation on this complex system. The albumin D-box binding protein (DBP) transcriptional activator, produced through the BMAL1-CLOCK loop, upregulates production of PER proteins. The nuclear factor interleukin-3 (NFIL3, alternatively known as E4BP4) repressor is regulated through a RORE. This factor acts to reduce PER protein production.

Though these transcriptional-translational feedback loops are the primary controllers of the circadian clock, posttranslational modification of this core machinery through the utilization of kinases, phosphatases, histone modifications, and epigenetic regulations provide supplementary regulatory mechanisms [8]. It has been shown that aberrations at this level in circadian control can have distinct phenotypes. For example, a missense mutation in the human *Per2* results in hypophosphorylation of its protein product by casein kinase 1 epsilon (CKIε), impaired degradation, and subsequent clinical manifestation as familial advance phase sleep disorder [9].

The aforementioned mechanisms are involved in preserving normal circadian rhythmicity. Both positive and negative transcriptional factors in these loops regulate expression of other clock-controlled genes (CCGs). CCGs themselves are proteins that have circadian rhythmicity but do not influence the core molecular machinery. These include proteins ranging from cell cycle proteins including c-Myc, p53, and p21 to inflammatory cascade proteins such as NF-κB [10].

## 3. Clock Machinery Mutations and Phenotypes from Genetic Studies

The majority of evidence regarding phenotypes resulting from circadian dysregulation stems from genetic studies where mutations in core clock proteins are introduced or through utilization of complete gene knockouts [11,12,13,14,15,16,17,18,19,20,21,22,23,24,25]. Therefore, many of these studies are descriptive in nature as they present phenotypes associated with specific clock machinery protein alterations. Though it is known that core circadian clock proteins have functions outside of the central circadian pathways [26,27], the degree to which these proteins do is poorly understood and prevents concrete establishment of a cause–effect relationship between gene mutation and the resulting phenotype. Given this, we will summarize a few of the findings resulting from genetic studies and the breakthroughs made regarding the attempt to correlate circadian disruption to clinical phenotypes. The main phenotypes we describe involve loss of circadian rhythmicity, increased tumor burden, and an accelerated rate of aging.

Previous research has shown *Clock/Clock* mutations in mice confer a lack of ability to sustain circadian rhythmicity [11]. The same finding has been shown with *Bmal1^−/−^* mice [12]. Consequently, it established the BMAL1 protein as the heterodimeric counterpart to CLOCK through demonstration of low levels of CLOCK controlled proteins, PER1 and PER2, in the mutant mice [12]. Furthermore, BMAL1’s potent role in preventing aging has been elucidated through showing that knockout mice tend to accumulate ROS and age prematurely (decreased life span, hair growth rate, and fat, bone, and muscle mass) [13]. While *Clock/Clock* mutant mice are more sensitive to genotoxic stressors, they do not exhibit an increased likelihood of tumor development on their own nor when exposed to low dose gamma radiation [14]. Rather, this mutation coupled with low dose gamma radiation induces aging. To our knowledge, no known association between increased likelihood for skin cancer development and *Bmal1* mutations has been established. In fact, *Bmal1^−/−^* mice have been shown to be less likely to develop skin tumors throughout initial, middle, and late stages of carcinogenesis [15]. *Bmal1^−/−^* mice crossed with a transgenic line expressing a strong oncogene had more differentiated squamous tumors compared to their wild type *Bmal1* counterparts. While the control group actually required euthanization by two months of age due to tumor burden, *Bmal1^−/−^* mice did not develop a comparable number or size of tumors [15].

In contrast to the above findings, *Cry1^−/−^Cry2^−/−^* mice’s lack of rhythmicity has been associated with constitutive transcriptional activity of CLOCK/BMAL1-controlled products [16,17]. Using these mice lines, it was shown that mutations in transcriptional activator proteins (CLOCK/BMAL1) lead to exquisite sensitivity to cyclophosphamide compared to their wild-type counterparts, while the repressor knockout mutants (*Cry1^−/−^Cry2^−/−^*) were significantly more resistant to the chemotherapeutic agent’s toxicity than were the wild type mice [18]. These results have conflicted with another study where *Cry1^−/−^Cry2^−/−^* mice and immortalized fibroblast lines showcased no substantial difference in survival rates compared to wild-type counterparts when exposed to both ionizing and UV radiation [19]. It was hypothesized here that mutation of this clock protein does not inherently confer a tumor-prone phenotype because of counterbalancing homeostatic mechanisms [19]. There exist other hypotheses for such disagreement in study results. While cell lines have been shown to maintain circadian rhythmicity of core clock genes *ex vivo* (*REV-ERB* *α* and *Per2*), they lose control of genes outside of this core circadian family [20]. This finding explains the opposing results of *Clock^−/−^*, *Bmal1*^−/−^, *Cry1^−/−^*Cry2^−/−^, or *Per1^−/−^Per2*^−/−^ cell lines showcasing no significant differences in DNA repair mechanisms when exposed to ionizing radiation, UV radiation, or chemotherapy induced DNA damage [21].

Period proteins exhibit a tumor suppressive role and mutations in these genes result in a tumor-phone phenotype as demonstrated through many murine models [22,23,24]. Highlighting the increased tumorigenesis resulting from loss of these genes, mice deficient in both functional *Per1* and *Per2* genes exhibit profound tendency for spontaneous development of lymphomas, hepatic tumors, and ovarian tumors [25].

The circadian control of such proteins is extremely complex and many definitive causational results have yet to be solidified. While there are multiple studies showcasing circadian rhythm proteins and their downstream effects, we chose to focus primarily on *in vivo* studies as it has been shown to better mimic clinical scenarios given loss of the circadian control of CCGs in immortalized cell lines [21].

## 4. Epidemiologic Studies of Shift Work and Carcinogenicity

Epidemiological studies examining circadian rhythm disruption could potentially be seen as a human correlate to the above *in vivo* studies examining alterations in clock machinery proteins. Such studies are generally performed in those engaging in shift work. The most comprehensive definition of a shift worker includes anyone working outside of 7:00 a.m.–6:00 p.m. work day [28], and it has been estimated that as many as 15% of Americans work during such non-standard hours [29]. In modern times, society has become industrialized with individuals spending the majority of their lives in man-made environments, surrounded by artificial lighting. Shift work, where individuals have atypical hours to maximize businesses’ productivity as well the public’s needs, is becoming increasingly common among a variety of professions [29]. The incidence of skin cancer has been extensively studied in several population-based studies in those participating in shift work including firefighters, nurses, and flight-based occupations—airline pilots and cabin crewmembers. Through studies on such groups, associations with shift work, its accompanying circadian rhythm disturbances, and a variety of cancers, including cutaneous malignancies, have been demonstrated [30,31]. There is thought that this work lifestyle likely contributes to a disruption of circadian rhythm and alters various homeostatic processes implicated in slowing tumorigenesis [32]. Thus, circadian disruption was classified by the International Agency for Research on Cancer as most probably carcinogenic based on evidence from prior human and animal studies [33]. We will discuss the skin cancer burden in each of these populations.

Firefighters often work in 24-h shifts which disrupts circadian rhythmicity [33]. Study results involving skin cancer incidence in this group have been variable, and it has only therefore been recognized that there exists a possible association between this occupation and melanoma (summary estimate risk of 1.32 (1.10–1.57)) [34]. More recent studies have highlighted this variability in findings. A recent analysis of cancer incidence in five Nordic studies demonstrated increased rates of melanoma in the 30–49 year cohort, while rates of non-melanoma skin cancer were only elevated in those 70 years and older [35]. This was again supported by another study of Scottish firefighters where melanoma incidence was statistically significantly elevated [36]. In contrast to these aforementioned findings, a low incidence of melanoma was demonstrated in a comparable group of firefighters ages 30–59 in another study [37]. There are multiple possible confounding factors to establishing a distinct link between skin cancers and circadian disruptions in this profession, one of which is the effect of environmental exposures. Firefighters are exposed to multiple potentially carcinogenic compounds. Environmental exposure from polycyclic aromatic hydrocarbons could likely contribute to the development of non-melanoma skin cancers in this group [38,39]. Further, trichloroethylene exposure could be potentially implicated in development of melanoma [40,41]. Due to the potential variability of chemical exposures internationally, it is difficult to establish how much circadian disruption interfaces with increased propensity for carcinogenicity. Assessment of firefighters’ sleep habits, shifts, and ambient exposures could prove helpful in ascertaining if circadian disruption contributes to tumor burden in this population.

In contrast, multiple studies have been concordant in showing a higher incidence of melanoma and other skin tumors in flight-based occupations [30,31]. UVR is one of the primary risk factors for skin carcinogenesis [42], but this risk factor may not be the cause of the increased cancer rates in airline crew as their exposure to solar UVB during flights is negligible [43] and there exists no statistically significant difference in UVR risk factors of crew compared to the general population despite more frequently vacationing in sunny regions [44]. Given this, alternate hypotheses have been examined for the increased skin tumor burden in pilots and cabin crewmembers. Flight-based occupations are exposed to higher levels of primary cosmic (ionizing) radiation including proton, neutron, electron, and gamma radiation. The International Commission on Radiological Protection recognizes that control of the exposure to the flight professionals is necessary as their radiation doses are similar to healthcare workers working with radiation [45]. The annual dose limit as recognized by this organization is 20 mSv [45], but it has been shown that aircraft pilots are well below this limitation receiving a median dose of 1.92 mSv yearly [46], which argues against suggestions of cosmic radiation as a possible reason behind the increased cancer burden in this group. A recent, robust meta-analysis of melanoma in airline pilots and cabin crew showed that the standardized incidence ratio (SIR) for airline cabin crew and pilots combined was 2.21 (95% CI, 1.76–2.77). SIR for pilots alone was 2.22 (95% CI, 1.67–2.93) and for cabin crew was 2.09 (95% CI, 1.67–2.62) [47]. In a subsequent article, the authors postulate that this increase in melanoma could be the result of UVA light penetration through cockpit windows as the UVA dose over an hour at this altitude is equivalent to that of twenty minutes spent in a tanning bed [48]. UVA light is able to penetrate through a variety of light colored fabrics, and this indeed may prove a potential explanation for this phenomenon [49]. This does, of course, not explain the increased incidence skin cancers in cabin crew. Resultant skin cancers are worrisome as standardized mortality ratio (SMR) analyses from a combined American and European cohort study demonstrate this as a significant cause of death in male pilots; this statistical significance was not found in male or female cabin crew [50]. It remains presently unclear whether the increased incidence of skin cancers in airline cabin crew and pilots results from disruptions in circadian rhythms, cosmic radiation, UVR dosage, or a combination of all these exposures.

Other shift workers, including nursing staff, have also been studied extensively to ascertain associations between various cancers and possible circadian disruption as well. To our knowledge, only one study in nursing staff has examined the effect of night shift work on skin carcinogenesis [51]. Schernhammer and associates ascertained that female nurse workers with over 10 years of time working night-shifts regularly were 14% less likely to develop skin cancer (for basal cell carcinomas, squamous cell carcinomas, and melanoma collectively) compared to those who had worked nightly for a short period of time or never [51]. With respect to melanoma specifically, it was shown that working during night-shifts bestowed a protective factor toward developing melanoma: A 44% decreased risk in development after adjusting for melanoma risk factors was shown [51].

Two other studies have examined skin cancer incidence rates in shift workers and found no significant association with skin cancer incidence [52,53]. The Schwartzbaum study identified 11 melanomas and six other non-melanoma skin cancers in the female group and 189 melanoma cases and 224 non-melanoma skin cancer in the male arm; these findings were not statistically significant nor different from expected rates [52]. Additionally, cancer rates were not elevated in men or women when taking all cancers combined [52]. It should be noted in this study that the authors postulate a few distinct reasons for finding no association between shift work and cancer. The authors did not control for risk factors other than marital status and socioeconomic status, their shift work definition was more restrictive, and study populations were comprised of large variety of professions. Similar findings and limitations have been identified in a cancer incidence study solely composed of men [53]. While there may be some evidence of a connection between shift work and skin cancer rates in certain populations, given the high number of confounding variables and that many epidemiological studies are focused primarily on identifying associations from exposures, definitive cause–effect relationships between circadian disruption and skin carcinogenesis cannot be established from available epidemiological data.

## 5. The Proposed Role of Melatonin on Skin Carcinogenesis

As circadian rhythm disruption in both murine and human studies may be linked to increased tumorigenesis, various hypotheses have been postulated and studies have been performed to explain the mechanism of this link. We will detail the work performed on characterizing the role of melatonin in skin carcinogenesis.

Focus on skin cancer and circadian dysrhythmia necessitates discussion of melatonin’s role in oncogenesis. The synthesis of melatonin, traditionally thought of as a pineal hormone, has been shown to oscillate in accordance with circadian rhythm in a 24-h pattern [54]. Melatonin’s role in cancer development was initially described in breast cancer. It was first theorized by Cohen and colleagues [55] that a reduction in function of the pineal gland, and by extension a relative lack of melatonin synthesis, would exhibit decreased inhibition on the synthesis of estrogens. Increased levels of estrogens have been linked to higher incidence rates of breast cancer, and therefore pineal dysfunction could be related to breast carcinogenesis [54,55]. This work began to lay the foundation for subsequent studies that set out to elucidate a relationship between melatonin and cancer.

Melatonin, however, is also synthesized at several peripheral sites ranging from bone marrow to gastrointestinal and skin cells [54,56]. This substance is protective against UVR-mediated photodamage through its scavenging of ROS and by exhibiting tumoristatic properties against tumors of epithelial origin [56,57]. The synthesis of melatonin is regulated by light; melatonin synthesis and release from the pineal gland increases at night [54], with light suppressing these actions [58]. Melatonin’s functional role in the skin as related to UVR is as follows. The major products of melatonin metabolism in the skin after UV radiation include *N*1-acetyl-*N*2-formyl-5-methoxykynuramine (AFMK), 2-hydroxymelatonin, 4-hydroxymelatonin, and 6-hydroxymelatonin. Of these products, AFMK is the most produced by UVR and forms in a dose-dependent and substrate dependent fashion [59]. AFMK is another strong free radical scavenger and is thought to contribute significantly to the prevention of DNA damage in human keratinocytes [60]. With circadian disruption, melatonin synthesis and secretion is altered. Therefore, dysregulation of the circadian clock decreases melatonin concentrations, reduces ROS scavenging by it and its products in the skin, and potentially promotes the formation of UVR-induced damage and accelerated carcinogenesis locally.

Melatonin’s light-based regulation prompted Stevens and associates [61] to theorize that the rise of breast cancer incidence seemed to follow the change in working conditions of society and the light-at-night (LAN) hypothesis was first established. The LAN hypothesis proposes that a decline in melatonin concentration decreases melatonin’s antioxidant and anti-carcinogenic activities. It was first seen in animal models that removal of the pineal gland increased the growth rates of melanomas [62]. Since then, studies showcasing absence or lack of melatonin exhibit an increased proliferative cellular state [63]. Conversely, administration of melatonin inhibits tumor growth in melanoma cells [57,64,65]. Associations between tumor progression and decreased levels of melatonin have been established. Rats exposed to dark periods with light contamination or exposed to 24 h of light either had blunted or completely absent nocturnal melatonin surges. When they were then implanted with subcutaneous Morris hepatoma line samples, tumor growth rates were negatively correlated with melatonin concentrations in the different exposure groups in a dose-response fashion [66].

Given that melatonin production exhibits circadian rhythmicity, the dysregulation of melatonin through disruption of circadian rhythm has been a proposed hypothesis to the carcinogenic effect of shift work [32]. It is implicated that LAN disrupts the normal circadian patters of melatonin synthesis and secretion, which in turn promotes neoplastic proliferation. The skin, specifically, due to a decreased synthesis of local melatonin, is likely more susceptible to the damaging effects of UVR and unable to repair the damage caused by ROS.

## 6. Circadian Control of UVR-Mediated DNA Damage

UVR is a primary risk factor for the development of a variety of skin cancers including melanoma [42]. UVR is implicated in carcinogenesis through both the creation of photoproducts by UVB and the formation of reactive oxygen species (ROS) by UVA. UVB causes direct damage of DNA through formation of cyclobutane pyrimidine dimers and pyrimidine (6-4) pyrimidone photoproducts that slow DNA replication, distort the DNA helix, and halt gene expression. Unrepaired dimers are highly mutagenic and are strongly implicated in skin carcinogenesis [42]. UVA produces ROS through a photochemical reaction involving the activation of various chromophores in the skin (porphyrins and tryptophan) that then react with oxygen to form the ROS. These ROS then either induce direct breakage of DNA strands or induce mutagenesis through the formation of the oxidized bases, 8-oxo-guanine or thymine glycol [42]. Though the carcinogenic effects of UVR on DNA in the skin are known, the direct effects of UVR on the skin’s autonomous circadian rhythm and the subsequent implications of this are less elucidated [8,67].

UVB irradiated keratinocyte cultures exhibit decreased expression levels of *Per1*, *Clock*, and *Bmal1* gene products initially [67]. Post irradiation, *Clock* and *Per1* gene expression recovered and eventually displayed increased mRNA levels; *Bmal1* expression, however, did not show any increase after recovery. While little data has been gathered examining ultraviolet radiation’s effects on clock machinery beyond this study, the converse situation is comparatively well studied. CCGs are involved in skin carcinogenesis prevention by modulating the response to photodamage through DNA repair [21,68,69,70,71,72].

Certain CCGs are intimately involved in tumor suppression; nucleotide excision repair activity manifested by the xeroderma pigementosum group A (XP-A) gene provides one example [68,69,73]. Nucleotide excision repair is mediated by six excision repair factors—XP-A, XP-C, XP-G (DNA damage-binding protein 2 (DDP2)), XPF (excision repair cross-complementation group 4 (ERCC4))–ERCC1 complex, transcription factor II H (TFIIH), and replication protein A (RPA)—that excise an oligomer. This gap is then filled in by DNA polymerases and ligases [68]. XP-A protein, specifically, is positively regulated by CLOCK-BMAL1 complex and negatively regulated by CRY and PER proteins; *Cry1^−/−^/Cry2^−/−^* mouse fibroblasts exhibit triple the level of XP-A expression levels relative to the wild type controls and elevated excision repair activity [69]. Further, Gaddameedhi *et al.* evaluated whether UV radiation at different time points affects skin cancer development. The authors discovered that mice irradiated during the early morning, when DNA repair is at a nadir, exhibited a five-fold increase in development of skin cancer compared to those in the evening [73].

Geyfman *et al.* [72] then demonstrated time-of-day dependent predisposition for developing UVR-induced ROS in a murine model that mirrored the initial findings of the Gaddameedi study. Because the murine circadian clock runs antiphase to the human clock [74], it is postulated that such findings suggest humans could have a higher rate of repair in the morning and be less prone to the carcinogenicity of UVR [72,73]. Clinically, sunburn incidence is a marker for the predisposition to develop skin tumors [42]. Findings that minimal erythema responses being greater in mice irradiated during the mornings again buttress the idea that timing of UVR exposure is implicated in carcinogenesis [70]. While interesting, it is still not understood if circadian regulatory mechanisms in humans are exact to that of mice. Therefore, epidemiological studies in humans assessing time-of-day sun exposure habits and the response to erythema with longitudinal follow-up might be considered so as to determine the role of UVR-time dependent exposure and its possible link to skin cancer development.

XP-A has been shown to follow a circadian rhythm, and it has been speculated that other DNA repair machinery may follow suit. DNA base excision repair via 8-oxo-guanine glycosylase (OGG1) is the primary means for removing 8-oxo-guanine photoproducts [75]. In a recent tri-component study, lymphocytes of 15 human subjects exposed to ROS-generating stressors displayed higher 8-oxo-guanine levels during the morning compared to the evening. During the evening, it should be noted that the increased presence of ROS species did not upregulate OGG1 expression suggesting that expression of the base excision repair enzyme is modulated by the endogenous circadian rhythm. This initial thought was buttressed in another *in vitro* study component demonstrating that *Bmal1^−/−^* human dermal fibroblasts lack oscillatory expression of OGG1 showcasing the effect of circadian disruption. However, this knock out line had increased OGG1 activity in response to DNA damage [71], which buttresses previous evidence of the tumor resistant nature of the *Bmal1^−/−^* mutation [15]. In support of the profound effects of circadian dysrhythmia on base excision repair, this same study found that the expression of OGG1 in lymphocytes from shift workers was significantly lower than in that of day workers [71]. Whether these associations are present similarly in skin cells remains poorly characterized.

## 7. Expression of Clock Proteins in Melanoma Biopsies

While the research presented above suggests some connections between skin oncogenesis and circadian rhythm, there is limited data examining clock machinery proteins within skin tumor specimens. Mice remain the model organism for elucidating variations in clock gene expression, given their well-developed genetics and relative general similarity to humans. It is established that human keratinocytes, melanocytes, dermal fibroblasts, and melanoma cells all display distinct, autonomous circadian rhythmicity, with differing frequency of circadian oscillation and different amplitudes of particular clock machinery gene expression shifts [22,67,76,77]. For example, human fibroblasts demonstrate faster oscillation (compared to keratinocytes and melanocytes) of clock machinery gene expression, while keratinocytes had higher amplitudes of oscillation [77]. However, in skin neoplasm specimens, there has been limited examination of core clock gene expression patterns. Lengyel performed a particular noteworthy study showing expression of clock genes in human skin tumors (melanoma) and tumor-adjacent skin [78]. These findings were then correlated with various histopathological and clinical characteristics to better understand these complicated molecular associations. It was found that *Per1*, *Per2*, *Clock*, and *Cry1* expression was reduced in the melanoma biopsies as compared to adjacent normal skin in the majority of melanoma patients. A negative correlation between PER1 protein expression and Breslow thickness was found but mRNA levels of *Per1* and *Per2* had no similar correlation. Both *Clock* mRNA and protein expression, on the other hand, showed a similar negative association. However, melanocytic nevi, benign skin tumors of melanocytes, showed similar findings to that of their malignant tumor counterparts: *Per2*, *Cry1*, and *Clock* genes all with decreased mRNA levels. Subsequent comparison between nevi and melanoma cells demonstrated no significant difference in *Per1* and *Per2* mRNA levels. Similarly, the number of PER1 immunopositive cells in melanoma as compared to nevus biopsies was not significant highlighting that *Per* expression is not inherently a feature of malignancy. Future studies would be of value to examine the clock machinery components’ expression and protein levels in melanoma specimens and in non-melanoma skin cancers. To further validate these findings, *in vitro* overexpression and knockdown experiments of specifically dysregulated clock machinery proteins in melanoma cell lines could help demonstrate whether individual proteins are critical for melanoma cell growth and viability. Additionally, crossing of melanoma mouse models with mice with disrupted clock machinery proteins, with subsequent observation of the rate and frequency of melanoma tumorigenesis, would be informative. This would help determine whether dysregulation of the clock machinery is directly involved in skin tumorigenesis or whether it is a symptom of tumor development, as previously discussed.

## 8. Conclusions

The data examining circadian dysregulation and the subsequent development of skin cancer is often contradictory with quite variable conclusions. Genetic studies involving knock out mutations of core clock proteins are not uniform in establishing a clinically significant phenotype from particular mutations. There are stronger associations with the oncogenic potential of some gene mutants (*Per*) than others (*Bmal1* and *Cry*). Further, epidemiologic studies provide conflicting results regarding the skin cancer burden in several populations suggesting that circadian disruption may only be one particular component in skin tumorigenesis. The circadian control of melatonin and, in particular, DNA repair mechanisms provides more robust evidence for supporting the link between circadian dysrhythmia and skin cancer development. Given the complexity and highly important nature of circadian clock proteins in concert with the paucity of literature on exact functions of the molecular clock, it is difficult to ascertain if there exist any definitive links between circadian rhythm disruptions and skin oncogenesis. The following investigations would yield a better understanding of what role clock machinery plays in skin tumor development: (1) further determination of the expression of clock genes in cutaneous tumors; and (2) alteration of clock machinery proteins or circadian rhythms in melanoma cell lines and mouse models with the subsequent determination of the effect on cell viability and/or tumor development, respectively.

## Abbreviations

SCN	Suprachiasmatic nucleus
BMAL1	Brain and muscle ARNT-like protein 1
CLOCK	Circadian Locomotor Output Cycles Kaput
PER	Period
CRY	Cryptochrome
ROR	Retinoic acid receptor-related orphan receptors
RORE	ROR response elements
DBP	Albumin D-box binding protein
NFIL3	Nuclear factor interleukin-3
CKIε	Casein kinase 1 epsilon
CCG	Clock-controlled genes
AFMK	*N*1-acetyl-*N*2-formyl-5-methoxykynuramine
LAN	Light-at-night
UVR	Ultraviolet Radiation
ROS	Reactive Oxygen Species
XPA	Xeroderma pigementosum group A
OGG1	8-oxo-guanine gycolylase
